# Decreased Bone Mineral Density Is Associated with Subclinical Atherosclerosis in Asymptomatic Non-Diabetic Postmenopausal Women

**DOI:** 10.3390/jcm14124033

**Published:** 2025-06-06

**Authors:** Jehona Ismaili, Afrim Poniku, Venera Berisha-Muharremi, Arlind Batalli, Rina Tafarshiku, Michael Y. Henein, Gani Bajraktari

**Affiliations:** 1 Clinic of Rheumatology, University Clinical Centre of Kosovo, 10000 Prishtina, Kosovo; jehonaismaili@yahoo.com; 2 Clinic of Cardiology, University Clinical Centre of Kosovo, 10000 Prishtina, Kosovo; arlind.batalli@uni-pr.edu (A.B.); gani.bajraktari@uni-pr.edu (G.B.); 3 Medical Faculty, University of Prishtina, 10000 Prishtina, Kosovo; venera.berisha@uni-pr.edu (V.B.-M.); rina.tafarshiku@uni-pr.edu (R.T.); 4 Clinic of Endocrinology, University Clinical Centre of Kosovo, 10000 Prishtina, Kosovo; 5 Imperial College London, London SW7 2AZ, UK; henein@gmail.com; 6 Siena University, 53100 Siena, Italy; 7 Department of Public Health and Clinical Medicine, Umeå University, 90187 Umeå, Sweden

**Keywords:** bone mineral density, osteoporosis, atherosclerosis, carotid ultrasound, menopauses

## Abstract

**Background/Objectives:** Estrogen deficiency is strongly related to osteoporosis, but its role in the development of atherosclerotic cardiovascular disease (CVD), particularly in postmenopausal women, is unclear. The aim of this study was to assess the relationship between osteopenia and subclinical atherosclerosis in asymptomatic non-diabetic postmenopausal women. **Methods:** This prospective study included 117 consecutive postmenopausal women (mean age 59 ± 7 years) referred from the outpatient Rheumatology Clinic of the University Clinical Centre of Kosovo, recruited between September 2021 and December 2022. Clinical, biochemical, bone mineral density (BMD), carotid ultrasound and coronary CT angiography data were analyzed. Subclinical atherosclerosis was diagnosed as the presence of carotid plaques and/or increased intima-media thickness (CIMT) > 1.0 mm. **Results:** Of the 117 studied women, 83 (71%) had osteopenia or osteoporosis (T-score < −1 SD), who had higher prevalence of carotid artery plaques (27.7 vs. 8.8%, *p* = 0.019), compared to those with normal BMD. They were, also, older (*p* < 0.001), had a longer duration of menopause (*p* = 0.004) and higher CAC scores (*p* < 0.019), compared to those without plaques. In multivariate analysis [odds ratio 95% confidence interval], age [1.244 (1.052–1.470), *p* = 0.001], osteoporosis [0.197 (0.048–0.806), *p* = 0.024] and CAC score > 10 HU [0.174 (0.058–0.806), *p* = 0.006] were independently associated with the presence of carotid plaques. **Conclusions:** Reduced BMD is highly prevalent in asymptomatic non-diabetic postmenopausal women and is associated with a high prevalence of subclinical carotid atherosclerosis. Age, osteoporosis and CAC score > 10 HU were independently associated with atherosclerotic carotid plaque formation. These findings highlight the potential pathophysiological link between osteoporosis and subclinical atherosclerosis.

## 1. Introduction

Cardiovascular diseases (CVDs) are the leading cause of death in women in the developed countries [1]. This threat increases significantly in post-menopausal women, although they lag behind men for developing acute vascular syndromes by 10 years [2]. The mechanism for the significant relationship between the risk of CVD and menopause remains uncertain [3]. Estrogen deficiency has been shown to have a strong relationship with osteoporosis [4], but its role in the development of CVD is unclear [5]. Postmenopausal women carry a significant risk of subclinical atherosclerosis, as shown by increased carotid intima–media thickness (CIMT) [6] and carotid plaque formation [7,8]. In elderly postmenopausal women, the decreased bone mineral density (BMD) has been shown to be associated with subclinical atherosclerosis and the occurrence of vascular events [9]. Several studies have reported a moderate inverse relationship between BMD and CIMT [10,11,12,13] as well as severity of coronary artery calcium (CAC) score [14,15], thus suggesting a potential relationship between osteoporosis and atherosclerosis.

Atherosclerosis risk factors are also well-established predictors of the development of CVD [16]. In particular, diabetes proved to be the most powerful predictor of subclinical atherosclerosis in postmenopausal women [17]. However, the association between BMD and subclinical atherosclerosis in non-diabetic postmenopausal women remains unclear. The aim of this study was to investigate the relationship between osteopenia and subclinical atherosclerosis in asymptomatic, non-diabetic postmenopausal women.

## 2. Methods

### 2.1. Patients

A total of 117 postmenopausal women (mean age 59 ± 7 years) were prospectively enrolled between September 2021 and December 2022 at the Outpatient Rheumatology Clinic, University Clinical Centre of Kosovo. Prior to participation, all individuals provided written informed consent. The study protocol received ethical approval from the Ethics Committee of the Medical Faculty at the University of Prishtina. All included patients have been in menopause for 9.5 ± 8 years, and underwent dual-energy X-ray absorptiometry (DEXA) scanning for suspicious osteoporosis after clinical examination by an experienced rheumatologist. Exclusion criteria included decompensated heart failure, active malignancy, hepatic or pulmonary disease, known coronary artery disease (CAD), diabetes and arterial hypertension.

### 2.2. Clinical Data

In all participants, demographic details, physical examination and anthropometric measurements were recorded. Body mass index (BMI) was calculated by dividing dry weight by body height (kg/m^2^). Blood pressure was measured using a brachial sphygmomanometer, after the subject had rested in the supine position for at least 10 min. The use of contraceptives and milk products were also documented.

### 2.3. Blood Analysis

All individuals underwent laboratory blood testing, including the following: hematology investigations, erythrocyte sedimentation rate, C-Reactive protein, fasting plasma glucose, blood urea nitrogen (BUN), creatinine, total cholesterol, LDL cholesterol, HDL cholesterol, triglycerides, total calcium, ionized calcium, anti-CCP, and vitamin D3 level. All blood samples were analyzed using standardized laboratory protocols, with duplicate testing and appropriate dilutions, following conventional methodologies.

### 2.4. Echocardiographic Examination

#### 2.4.1. Cardiac Structure and Function

The echocardiographic examination was performed by a single operator, using a Philips Intelligent E-33 system with a multi-frequency transducer. The patient was in the left lateral decubitus position during the obtaining of images. The LV dimensions at end-diastole and end-systole, as well as interventricular septal and posterior wall thickness, were measured according to the Guidelines of the American Society of Echocardiography and the European Association of Cardiovascular Imaging [18,19]. The volumes of LV and its ejection fraction (EF) were measured and calculated from the apical 2 and 4 chamber views using the modified Simpson’s method. The severity of mitral regurgitation was assessed by color and continuous-wave Doppler and it was graded according to the American Society of Echocardiography and the European Association of Cardiovascular Imaging as mild, moderate or severe [20,21]. The color Doppler and continuous-wave Doppler were used to assess tricuspid regurgitation. Pulmonary hypertension was evidenced if the retrograde trans-tricuspid pressure drop was >35 mmHg [22].

#### 2.4.2. Carotid Ultrasound Measurements

A 12-3-MHz Esaote Biomedica MyLab40 Ultrasound System was used to assess the carotid arteries, with all images acquired by a single expert operator. The distal portion of the common carotid artery was examined to measure CIMT, and plaques were identified according to the Mannheim consensus criteria, defined as focal thickenings extending into the lumen by at least 0.5 mm or exhibiting a total wall thickness greater than 1.5 mm, measured from the interface between the intima and lumen to the boundary between the media and adventitia [23,24]. The distal segments of both right and left common carotid arteries (CCAs) were the segments used to measure CIMT. Carotid atherosclerosis (CA) was identified as CIMT > 1 mm and/or the presence of plaques. To determine CIMT values, a semi-automated software (radiofrequency-based software-guided technique quality intima–media thickness—RF-QIMT-version 7.1.1, Esaote, Genoa, Italy) was used, after recording and subsequently analyzing images. The arithmetic average of the maximum left and right common carotid IMT was defined as a composite measure of CIMT.

#### 2.4.3. Coronary CT Angiography

Coronary imaging was conducted using a 64-slice multidetector CT scanner (Siemens Somatom Volume Zoom) with a gantry rotation time of 330 milliseconds. The scanning parameters included a collimation of 64 × 0.6 mm, a reconstruction interval of 0.3 mm, and a tube voltage of 100 kV. Image acquisition was carried out during a 10 s inspiratory breath-hold. To control heart rate, patients with resting rates above 65 beats per minute received an oral dose of metoprolol (50–100 mg) approximately one hour before the scan. For detection of coronary artery calcification (CAC), a non-enhanced scan was performed. The prospective ECG triggering was used and the scan was usually performed at 70% of the RR interval. The collimation was set to 30 × 0.6 mm, and the reconstructed slice thickness was 3 mm (adapted field of view depending on heart size, matrix 512 × 512, pixel size usually 0.5 × 0.5 mm). The CAC score calculation was performed using the Agatston method, determined by the calcified area and CAC density [25]. The software package (‘SyngoCaScore’, version VB10, Siemens Healthcare, Forchheim, Germany) was used. The presence of >2 contiguous pixels with >130 Hounsfield units was defined as presence of calcium, and these lesions were automatically identified and marked in color by the workstation. The total CAC score was calculated as the sum of calcium scores in all branches of coronary arteries. The CAC score was measured by an observer blinded to the coronary angiogram results and clinical data.

#### 2.4.4. Measurement of Bone Mineral Density (BMD)

BMD was measured in the lumbar spine and in the right and left femoral neck using dual-energy X-ray absorptiometry (Lunar DPX NT-400157; GE Healthcare, Madison, WI, USA). The BMD was measured in all study subjects assessing 4 lumbar vertebrae (L1–L4) and the central portion of a lateral scout view of the 1st to the 4th vertebra. The BMD of participants were categorized into normal (T-score ≥ −1 SD) or low (osteopenia, T-score between −1 and −2.5 SD/osteoporosis, T-score ≤ −2.5 SD) [26]. The standard protocols, provided by the manufacturers, were used for all measurements, which were performed by the same experienced operator.

### 2.5. Statistical Analysis

We tested the normality of continuous variables using the Shapiro–Wilk test. Variables were expressed as means ± standard deviation (SD) if normally distributed and as the median (Q1–Q3) if not normally distributed, and were compared using the Mann–Whitney U test. Group differences were analyzed using the unpaired Student *t* test for continuous variables. Pearson correlations were performed to identify simple correlations between variables. The Chi-square test was used to compare the categorical variables. *p* values < 0.05 were considered statistically significant. All analyses were performed using SPSS 22 for windows.

## 3. Results

### 3.1. Clinical and Biochemical Data in Women with and Without Osteopenia/Osteoporosis (Table 1)

Of the 117 included postmenopausal women, 83 (71%) had osteopenia or osteoporosis (T-score < −1 SD). These individuals had a higher percentage of carotid artery plaques (27.7 vs. 8.8%, *p* = 0.019), compared to those with normal BMD (Table 1). All other clinical, demographic, risk factors and biochemical data did not differ between these two groups. In addition, CAC score was not significantly different between postmenopausal women with and without osteopenia or osteoporosis.

**Table 1:** Clinical and biochemical data in postmenopausal women.

Variable	Normal	Osteopenia- Osteoporosis	*p* Value
	(n = 34)	(n = 83)	
Age (years)	58 ± 5	59 ± 7	0.347
Years of menopause [Median (Q1–Q3)]	7 (2–10)	8 (4–15)	0.060
Smoking (%)	21.7	7.5	0.060
Body-mass index (kg/m^2^)	27.4 ± 5	29.3 ± 5	0.104
Erythrocyte sedimentation rate (mm/h) [Median (Q1–Q3)]	22 (12–32)	11 (20–27)	0.484
C-Reactive protein (%)	7 ± 8	7.1 ± 7	0.928
Total calcium (mmol/L)	2.3 ± 0.2	2.3 ± 0.2	0.410
Ionized calcium (mmol/L) [Median (Q1–Q3)]	1.45 (1.2–1.8)	1.3 (1.2–1.8)	0.775
Vitamin D3 (IU)	22.5 ± 6	24 ± 14	0.648
Anti-CCP (u/mL) [Median (Q1–Q3)]	8 (6.8–8)	7 (6.7–8)	0.366
Glucose (mmol/L)	5.3 ± 1.0	5.3 ± 0.8	0.715
Total cholesterol (mmol/L)	5.4 ± 0.7	5.5 ± 1.0	0.875
Triglycerides (mmol/L)	1.8 ± 0.5	1.7 ± 0.6	0.327
HDL cholesterol (mmol/L)	1.4 ± 0.4	1.4 ± 0.3	0.816
LDL cholesterol (mmol/L)	3.0 ± 0.7	3.4 ± 0.8	0.527
Creatinine (μmol/L) [Median (Q1–Q3)]	78 (70–85)	76 (66–87)	0.624
Urea (mmol/L) [Median (Q1–Q3)]	5.45 (4.5–7.6)	5.6 (4.5–7.0)	0.978
Hemoglobin (g/dL)	123 ± 17	125 ± 13	0.707
Heart rate at admission (beats/min)	65 ± 12	68 ± 10	0.197
Use of contraceptives (%)	13	11.7	0.549
Using milk products (%)	91.3	92.5	0.567
Patients with CAC (%)	73.9	79.8	0.359
CAC score (HU) [Median (Q1–Q3)]	1.55 (00–31)	1.0 (0.1–31)	0.811

Anti-CCP: anti-cyclic citrullinated peptide; HDL: high-density lipoprotein; LDL: low-density lipoprotein; CAC: coronary artery calcium.

### 3.2. Cardiac Structure and Function in Women with and Without Osteopenia/Osteoporosis (Table 2)

In postmenopausal women with osteopenia or osteoporosis, transmitral E wave deceleration time was shorter (*p* = 0.037), septal wall e’ velocity was higher (*p* = 0.025) and the left atrium was larger (*p* = 0.019), compared to those without osteopenia or osteoporosis. All other cardiac structure and function measurements assessed by Doppler echocardiography were not different between the two groups of women, with and without osteopenia or osteoporosis (Table 2).

**Table 2:** Echocardiographic data in postmenopausal women.

Variable	Normal (n = 34)	Osteopenia- Osteoporosis (n = 83)	*p* Value
*Echocardiographic Data*			
Inter ventricular septum (cm)	10.3 ± 1.3	10.4 ± 2.5	0.765
LV posterior wall (cm)	9.6 ± 1.6	9.9 ± 1.2	0.309
LV end-diastolic diameter (cm)	46 ± 4	48 ± 6	0.234
LV end-systolic diameter (cm) [Median (Q1–Q3)]	30 (27–34)	31 (29–33)	0.113
*LV systolic function*			
LV ejection fraction (%) [Median (Q1–Q3)]	63 (55–70)	63 (58–67)	0.532
LV shortening fraction (%) [Median (Q1–Q3)]	34.5 (30.5–40)	35 (30.8–39)	0.781
*Carotid Ultrasound Data*			
Maximal CIMT (cm) [Median (Q1–Q3)]	0.09 (0.08–0.11)	0.09 (0.08–0.11)	0.615
Maximal CIMT ≥ 1 mm (%)	37.3	38.2	0.482
Presence of carotid plaque (%)	8.8	27.7	0.019

LV: left ventricular; CIMT: carotid intima–media thickness.

### 3.3. Clinical and Biochemical Data in Women with and Without Atherosclerotic Plaques (Table 3)

Postmenopausal women with atherosclerotic plaques, detected by carotid ultrasound examination, were older (*p* < 0.001), had a longer duration of menopause (*p* = 0.004) and had a higher CAC score (*p* < 0.019) compared to those without atherosclerotic plaques. All other clinical and biochemical data did not differ between these two groups (Table 3).

**Table 3:** Clinical and biochemical data in postmenopausal women with and without atherosclerotic plaques.

Variable	Without Plaques	With Plaques	*p* Value
	(n = 91)	(n = 26)	
Age (years) [Median (Q1–Q3)]	57 (54–61)	62.5 (60–65)	<0.001
Years of menopause (years) [Median (Q1–Q3)]	6 (3–14)	13.5 (8–19)	0.770
Smoking (%)	8.8	15.4	0.330
Body-mass index (kg/m^2^)	29 ± 5	28.7 ± 4	0.757
Erythrocyte sedimentation rate (mm/h)	21 ± 14	23 ± 12	0.472
C-Reactive protein (%) [Median (Q1–Q3)]	6 (2–8)	4.1 (2–11.5)	0.724
Total calcium (mmol/L) [Median (Q1–Q3)]	2.4 (2.1–2.5)	2.3 (2.2–2.4)	0.842
Ionized calcium (mmol/L) [Median (Q1–Q3)]	1.4 (1.2–1.8)	1.3 (1.2–1.7)	0.653
Vitamin D3 (IU)	24.8 ± 14	21 ± 8	0.081
Anti-CCP (u/mL) [Median (Q1–Q3)]	7 (6–8)	8 (7–8)	0.044
Glucose (mmol/L)	5.4 ± 0.8	5.2 ± 1.0	0.571
Total cholesterol (mmol/L)	5.4 ± 1.0	5.7 ± 0.7	0.110
Triglycerides (mmol/L)	1.7 ± 0.6	1.8 ± 0.5	0.487
HDL cholesterol (mmol/L)	1.4 ± 0.4	1.4 ± 0.2	0.816
LDL cholesterol (mmol/L)	3.0 ± 0.8	3.4 ± 0.6	0.528
Creatinine (μmol/L)	77 ± 19	81 ± 18	0.891
Urea (mmol/L) [Median (Q1–Q3)]	5.5 (4.5–7)	5.6 (4.5–7)	0.864
Hemoglobin (g/dL)	125 ± 14	123 ± 12	0.473
Use of contraceptives (%)	13.2	7.7	0.448
Using milk products (%)	90	100	0.205
CAC score (HU) [Median (Q1–Q3)]	0.4 (00–8.1)	30 (1.3–150)	<0.001

Anti-CCP: anti-cyclic citrullinated peptide; HDL: high-density lipoprotein; LDL: low-density lipoprotein; CAC: coronary artery calcium.

### 3.4. Correlates of Carotid Plaque Formation in Postmenopausal Non-Diabetics (Table 4)

In the univariate analysis model, age (*p* = 0.001), menopause years (*p* = 0.005), presence of osteoporosis (*p* = 0.035), CAC score (*p* = 0.010), and CAC score > 10 HU (*p* = 0.001) were associated with the presence of carotid plaques. In multivariate analysis [odds ratio 95% confidence interval], only age [1.244 (1.052–1.470), *p* = 0.001], the presence of osteoporosis [0.197 (0.048–0.806), *p* = 0.024] and CAC score > 10 HU [0.174 (0.058–0.806), *p* = 0.006] proved to be independently associated with the presence of atherosclerotic carotid plaques in the studied cohort (Table 4).

**Table 4:** Correlates of the presence of atherosclerotic plaques in postmenopausal non-diabetic women.

	Univariate Analysis			Multivariate Analysis		
Variable	OR	CI 95%	*p*	OR	CI 95%	*p*
Age	1.145	(1.060–1.236)	0.001	1.244	(1.052–1.470)	0.011
Menopause (years)	1.085	(1.025–1.148)	0.005			
Osteoporosis	3.961	(1.103–14.23)	0.035	4.435	(1.121–17.55)	0.024
CAC score	1.005	(1.001–1.009)	0.013			
CAC score > 10	4.987	(1.968–12.64)	0.001	4.604	(1.684–12.86)	0.006

CAC: coronary artery calcium.

## 4. Discussion

*Findings*: In this study of a group of non-diabetic asymptomatic postmenopausal women, our analysis showed the following: (1) decreased bone mineral density, expressed as osteopenia or osteoporosis, is highly prevalent (71%) in asymptomatic non-diabetic postmenopausal women; (2) postmenopausal women with osteopenia or osteoporosis had higher prevalence of carotid artery plaques, compared to those with normal BMD; (3) postmenopausal women with atherosclerotic plaques had higher CAC score; and (4) age, the presence of osteoporosis and CAC score > 10 HU were independently associated with the presence of atherosclerotic carotid plaques.

*Data interpretation*: Our findings support the hypothesis of a link between osteoporosis and subclinical atherosclerosis, suggesting that bone loss and vascular calcification may share common pathological pathways. The 71% prevalence of low BMD in our cohort is consistent with prior reports which showed postmenopausal women are at a high risk for bone loss due to estrogen deficiency [27]. It is well established that estrogen plays a key role in maintaining bone mass and in having a protective effect for the cardiovascular system. Its deficiency has been shown to accelerate both bone resorption and vascular aging [28]. Thus, the higher prevalence of carotid plaques in women with osteopenia/osteoporosis, we have found, suggests a shared etiology between osteoporosis and vascular calcification. Studies have previously proposed that osteoporotic bone loss and vascular calcification may be driven by chronic inflammation, oxidative stress, and dysregulated calcium-phosphate metabolism [29]. Our results align with these studies in showing an inverse relationship between BMD and carotid intima–media thickness (CIMT) as well as coronary artery calcification (CAC) scores [30]. The significant association between carotid plaques and higher CAC scores suggests that atherosclerosis is a systemic process affecting both carotid and coronary arteries [31], although mature phenotypic presentation might differ between the two arterial systems. CAC is an established marker of subclinical atherosclerosis and future cardiovascular events [32], further supporting the clinical importance of early vascular screening in postmenopausal women. Finally, our multivariate analysis revealed that age, osteoporosis, and CAC score > 10 HU were independent correlates with carotid plaques. Age has an established strong correlation with atherosclerosis [33]; such a relationship is likely to be due to prolonged exposure to cardiovascular risk factors [34]. The independent association between osteoporosis and carotid plaques further supports the hypothesis of bone–vascular crosstalk [35].

*Clinical implications*: Postmenopausal women, particularly those with low BMD, may benefit from early vascular assessment to identify subclinical atherosclerosis. Osteoporosis and atherosclerosis may share common inflammatory and metabolic pathways, warranting multidisciplinary management strategies. Future research should explore whether bone-protective therapies (e.g., bisphosphonates, vitamin D, calcium) could mitigate vascular calcification risk.

*Study limitations*: The sample volume of this study is modest, which limited sub-analyses which could have identified potential differences between patients with osteopenia and osteoporosis. This is a single-center study and these findings may not be generalizable to other populations. Another important limitation is that factors such as diet, physical activity, and inflammatory markers were not assessed. Future prospective studies should investigate whether bone-targeted interventions impact vascular outcomes in postmenopausal women.

## 5. Conclusions

Reduced bone mineral density is highly prevalent in asymptomatic non-diabetic postmenopausal women and is related to subclinical atherosclerosis. Our findings suggest that osteoporosis and vascular disease may be linked through shared pathophysiological mechanisms, yet to be determined. Age, osteoporosis, and coronary calcification were independently associated with carotid atherosclerosis, thus underscoring the need for early screening and potential targeted prevention strategies in postmenopausal women.

## Data Availability

Data are available from the corresponding author upon reasonable request.
